# Study on the ability of 3D gamma analysis and bio-mathematical model in detecting dose changes caused by dose-calculation-grid-size (DCGS)

**DOI:** 10.1186/s13014-020-01603-6

**Published:** 2020-07-06

**Authors:** Han Bai, Sijin Zhu, Xingrao Wu, Xuhong Liu, Feihu Chen, Jiawen Yan

**Affiliations:** Department of Radiation Oncology, Yunnan Tumor Hospital, The Third Affiliated Hospital of Kunming Medical University, No.519 Kunzhou, Road, Xishan District, Kunming, Yunnan China

**Keywords:** 3D gamma analysis, Bio-mathematical model, Dose calculation grid size (DCGS), Dose change

## Abstract

**Objective:**

To explore the efficacy and sensitivity of 3D gamma analysis and bio-mathematical model for cervical cancer in detecting dose changes caused by dose-calculation-grid-size (DCGS).

**Methods:**

17 patients’ plans for cervical cancer were enrolled (Pinnacle TPS, VMAT), and the DCGS was changed from 2.0 mm to 5.0 mm to calculate the planned dose respectively. The dose distribution calculated by DCGS = 2.0 mm as the “reference” data set (RDS), the dose distribution calculated by the rest DCGS as the“measurement”data set (MDS), the 3D gamma passing rates and the (N) TCPs of the all structures under different DCGS were obtained, and then analyze the ability of 3D gamma analysis and (N) TCP model in detecting dose changes and what factors affect this ability.

**Results:**

The effect of DCGS on planned dose was obvious. When the gamma standard was 1.0 mm, 1.0 and 10.0%, the difference of the results of the DCGS on dose-effect could be detected by 3D gamma analysis (all *p* value < 0.05). With the decline of the standard, 3D gamma analysis’ ability to detect this difference shows weaker. When the standard was 1.0 mm, 3.0 and 10.0%, the *p* value of > 0.05 accounted for the majority. With DCGS = 2.0 mm being RDS, ∆gamma-passing-rate presented the same trend with ∆(N) TCPs of all structures except for the femurs only when the 1.0 mm, 1.0 and 10.0% standards were adopted for the 3D gamma analysis.

**Conclusions:**

The 3D gamma analysis and bio-mathematical model can be used to analyze the effect of DCGS on the planned dose. For comparison, the former’s detection ability has a lot to do with the designed standard, and the latter’s capability is related to the parameters and calculated accuracy instrinsically.

## Introduction

VMAT technology is widely used for the cervical cancer radiotherapy [1–3]. These studies on the application have shown that VMAT technology can be used for cervical cancer radiotherapy, and compared with High Dose Rate brachytherapy, VMAT plan achieves significant dose reduction of rectum, bladder and sigmoid, as well as superior homogeneous target coverage compared to brachytherapy plan [4–6].

The dose calculation grid size (DCGS) is a basic parameter setting in the design of the plan. Usually, a commercial treatment planning system (TPS) will provide various DCGS within a certain range for designers to choose for different needs. For example, the commercial Pinnacle TPS provides DCGS ranging from 1.0 mm to 10.0 mm, and the default DCGS is 4.0 mm [7]. Larger DCGS is commonly adopted for calculation in cases with larger target volumes and organ-at-risk (OAR) volumes for better calculation efficiency. However, a smaller DCGS should be chosen for dose calculation in radiotherapy for head and neck tumors to obtain precise doses of small-volume OARs including lens, optic nerve and pituitary, etc., especially important for OARs with strict maximumdose limit [8, 9].

The difference in doses caused by different grid sizes may affect the evaluation of the quality of physical solutions, although the DCGS can not cause the actual absorbed dose when the accelerator’s parameters are certain. (This is why the planned dose values and (N) TCP values calculated under different DCGS are all called “calculated” values in the following sections). Therefore, it is a crucial task to understand the effect of DCGS on the physical and biological doses in radiotherapy for cervical cancer. Gamma analysis is currently the most common and generally accepted method for quantitatively assessing the difference between the two dose-distribution (DD) [10, 11]. It detects the difference between the two DD by a designed gamma standard (e.g. 3.0 mm, 3.0, 10%) and it will provide a report on passing rate [12, 13]. The standard of 3.0 mm, 3.0 and 10% is the most widely used, in which 3.0 mm refers to the consistency of distance, 3.0% refers to the maximum allowable dose difference, and 10% is the threshold (In the following parts of the article we skipped it as it never changed) and when the dose is less than 10% of the reference dose, which does not participate in gamma analysis. Selecting 10% is widely recommended [14, 15]. In intensity modulated radiation therapy (IMRT), gamma analysis is usually used to analyze the difference between the TPS-outputed and the actually measured dose distribution to evaluate the degree of dose deviation caused by various reasons during the execution of the plan, further to determine whether a plan is to execute based on the evaluation. However, previous studies have shown that different dose quality assurance (QA) system (dose QA system refers to the collection of measurement and analysis software and hardware used to ensure that the radiation dose is achieved at the target volume and the OARs as expected) have different abilities to detect errors based on the dose distribution output by TPS. Hussein et al. [16] enrolled pelvis and head & neck IMRT and RapidArc™ plans, and compared the differences in the detecting dose error of five commercial products: PTW Verisoft, Delta4 software, SNC Patient, Varian Portal Dosimetry and IBA OmniPro. The results showed that for the same pass-rate criteria, different devices and software combinations exhibited varying levels of agreement with the predicted gamma analysis. On the other hand, different gamma analysis standards will get different passing rates. Research by Heilemann et al. [17] showed that the 3.0 mm, 3.0% standards were not sufficient to detect the deviation caused by the MLC position uncertainty, and this standard, at least, has to be 2.0 mm, 2.0%.

The focus on the variation of planned dose (PD) is due to the fact that it could cause changes in the biological effects. Specifically, in the clinical practice of radiotherapy, the alteration in physical dose will bring about changes in tumor control probability (TCP) and nomal tissue complication probability (NTCP). Therefore, the current project, under the condition of DCGS changes, used the dose distribution calculated by DCGS = 2.0 mm as the reference data set (RDS) to explore the efficacy and sensitivity of the 3D gamma analysis and the bio-mathematical model on dose change detection by analyzing the 3D gamma passing rates of all structures and the relationship between gamma passing rate and (N)TCP.

## Materials and methods

### Patient meterials

A retrospective study was performed on the physical plan of 17 patients with cervical cancer who were treated in the Department of Radiation Therapy of our hospital from December 2017 to November 2018. And the 17 patients’ plans were intially designed and evaluated with DCGS = 4.0 mm. The patient’s planning gross target volume (PGTV) volume was 20.0–395.0cm^3^, and the planning target volume (PTV) volume was 880.0–2587.0cm^3^. The average volumes of the two target volume were 128.9 ± 110.2 cm^3^, 1752.9 ± 460.1 cm^3^, respectively. The rectum’s mean volume was 59.3 ± 25.4 cm^3^, and the bladder’s was 257.5 ± 165.6 cm, and the L-femur’ s and R-femur’s were 107.2 ± 19.1 cm^3^, 108.2 ± 19.6 cm^3^. These patients were in a supine position with both hands surrounding their heads, and the patient was fixed with a thermoplastic mesh. The Siemens Somatom Sensation Open 24 CT (Siemens Co., Munich, Germany) was used as the data acquisition system. The range of scanning was from the head of the diaphragm to lower 1.0 cm of the bottom pubic symphysis. And the CT data of each patient was reconstructed with a 3.0 mm layer thickness, was transmitted to Pinnacle TPS 9.10.

### Design of volumetric modulated arc therapy (VMAT) radiotherapy plan

17 patients were treated with a Versa HD linear accelerator (Elekta Medical Systems Co., Stockholm, Sweden) of 6 MV photon beams. The VMAT plan of a 360^o^ full bow with 2 arcs was designed for every patient based on Smart Arc inverse optimization. The objective functions were shown in Table 1. The doses were calculated with the Collapsed Cone Convolution (CCC) algorithm [18]. The planning prescription setting was as follows: the PTV prescription being 45.0–50.0Gy/25 fractions, and the PGTV prescription being 60.0–62.5Gy/25 fractions.

**Table 1:** Dose-volume criteria used in the cervix cancer VMAT plans

Volume of interest	Dose-volume criteria (cGy)
PGTV	MinD = 95%PD, V_PD_ ≥ 95%, MaxD = 107%PD
PTV	MinD = 95%PD, V_PD_ ≥ 95%, MaxD = 107%PD
Rectum	V40 < 60%, D_33%_ < 45Gy
Bladder	V40 < 40%, D_33%_ < 45Gy
L-Femur	V45 < 5%, V30 < 30%
R-Femur	V45 < 5%, V30 < 30%
Intestine	V30 < 30%
Cord	MaxD = 45Gy

Note: PD is the prescribed dose

All VMAT physical schemes were designed with Pinnacle TPS (version 9.10). When the default value was DCGS = 4.0 mm, the planners optimized and adjusted the treatment plans for cervical cancer patients based on their own previous experience. After all the indicators of the plans met the clinical requirements, changed the DCGS (from 2.0 mm to 5.0 mm) and recalculated dose in the target volumes and OARs.

#### D gamma standard and passing rate

After the emergence of IMRT technology, verification of radiation dose before the implementation of treatment has become a very important part of the radiotherapy process. Dose verification can be divided into point dosimetry verification, plane dosimetry verification and gel dosimetry verification. The point dosimetry verification and Gel dosimetry verification [19, 20] have not been widely accepted because of various reasons, and the plane dosimetry verification has become a popular method. The commercially available PTW-ARRAYs [21] and IAB-ARRAYs [22] are the most popular tools for plane dose verification. These ARRAYs only respond correctly to beams perpendicular to their matrix plane, and it is necessary to combine the scattered beams into one direction when using these ARRAYs. This feature makes these ARRAYs less suitable for dose verification of VMAT with rotating beams. So, Delta 4, ArcCheck and Octavius 4D for dose verification of VMAT are more common in daily work [23–25].

Although the tools advanced, there has been no fundamental change in analytical methods. The gamma analysis has been used throughout IMRT dose verification. The commonly recommended gamma analysis standards are 3.0 mm, 3.0% [14, 15], but studies have shown that, depending on the technology and the disease, we should adopt stricter standards or other supplement analysis to analyze errors [26, 27].

The 3D gamma analysis is also a standard gamma analysis. The 3D gamma analysis is that during the dose of reconstruction before the analysis of gamma analysis, the software will be used the planned dosed Perturbation (PDP) algorithm corresponding the hardware. For example, if the hardware is ArcCHECK, the corresponding algorithm is ArcCHECK-planned-dosed-perturbation (AC-PDP) [28].

Although both are gamma analysis, there are differences between 2D gamma analysis and 3D gamma analysis in the range of detection analysis (2D dose is extracted from the 3D dose at the isocenter position) and sensitivity of the error detection [15].

This article was to investigate errors among planned dose caused by DCGS with dose distribution calculated by DCGS = 2.0 mm as RDS and the dose distribution calculated by DCGS = 3.0 mm, 4.0 mm, 5.0 mm as MDS, respectively. And we thought the location uncertainty was scarcely influential element under the situation. Therefore, when setting the gamma analysis standard, we setted the following 3 standards: 1.0 mm, 1.0%; 1.0 mm, 2.0%; 1.0 mm, 3.0%.

### TCP and NTCP calculation

The link between physical dose (change) and biological effect (change) has always been our focus. Changes in DCGS will certainly bring about changes in the physical dose, as well as the biological effects. It is well known that changes in biological effects have more direct clinical significance [29], so this study ignored the physical dose and directly calculated the changes in biological effects caused by changes in DCGS by using biological model.

Some biologically related models for plan optimization and/or evaluation have been introduced into treatment planning tools for clinical use. A variety of dose- response models with a series of organ-specific model parameters were reported in the literatures, and were widely accepted as follows [30, 31]:

1 $$ \mathrm{TCP}=\frac{1}{1+{\left(\frac{{\mathrm{TCD}}_{50}}{\mathrm{EUD}}\right)}^{4\gamma 50}} $$

2.1 $$ \mathrm{NTCP}=1/\sqrt{2\pi }{\int}_{-\infty}^u{e}^{-{t}^{\raisebox{1ex}{$2$}\!\left/ \!\raisebox{-1ex}{$2$}\right.}} dt $$

2.2 $$ u=\left(\mathrm{EUD}\hbox{-} {\mathrm{TD}}_{50}\right)/\left({\mathrm{mTD}}_{50}\right) $$

2.3 $$ {\mathrm{TD}}_{50}(v)={\mathrm{TD}}_{50}(1)\ast {v}^{-n} $$

2.4 $$ \mathrm{EUD}={\left({\sum}_i{v}_i{\mathrm{D}}_i^a\right)}^{1/a} $$

Where, *a* is an organ-specific constant, and its corresponding value is in the literatures [32, 33]. *v*_*i*_ is the fractional volume of the organ receiving *D*_*i*_. *m* and *n* are unique organ-specific constants [32, 33]. TD_50_ is an uniform dose that is absorbed dose at a 50% complication probability, and TCD_50_ is an uniform dose that is absorbed dose at a 50% control probability.

### Statistical analysis

Origin 8.0 was used for drawing and SPSS 20.0 was used for statistical analysis. *Paired t test* was used for statistical analysis, and *p > 0.05* indicates no significant difference, and *0.01 < p < 0.05* indicates significant difference, and *p < 0.01* indicates very significant difference.

## Results

### (N) TCP and absolute dose (AD) changes with DCGS

**Fig. 1:**
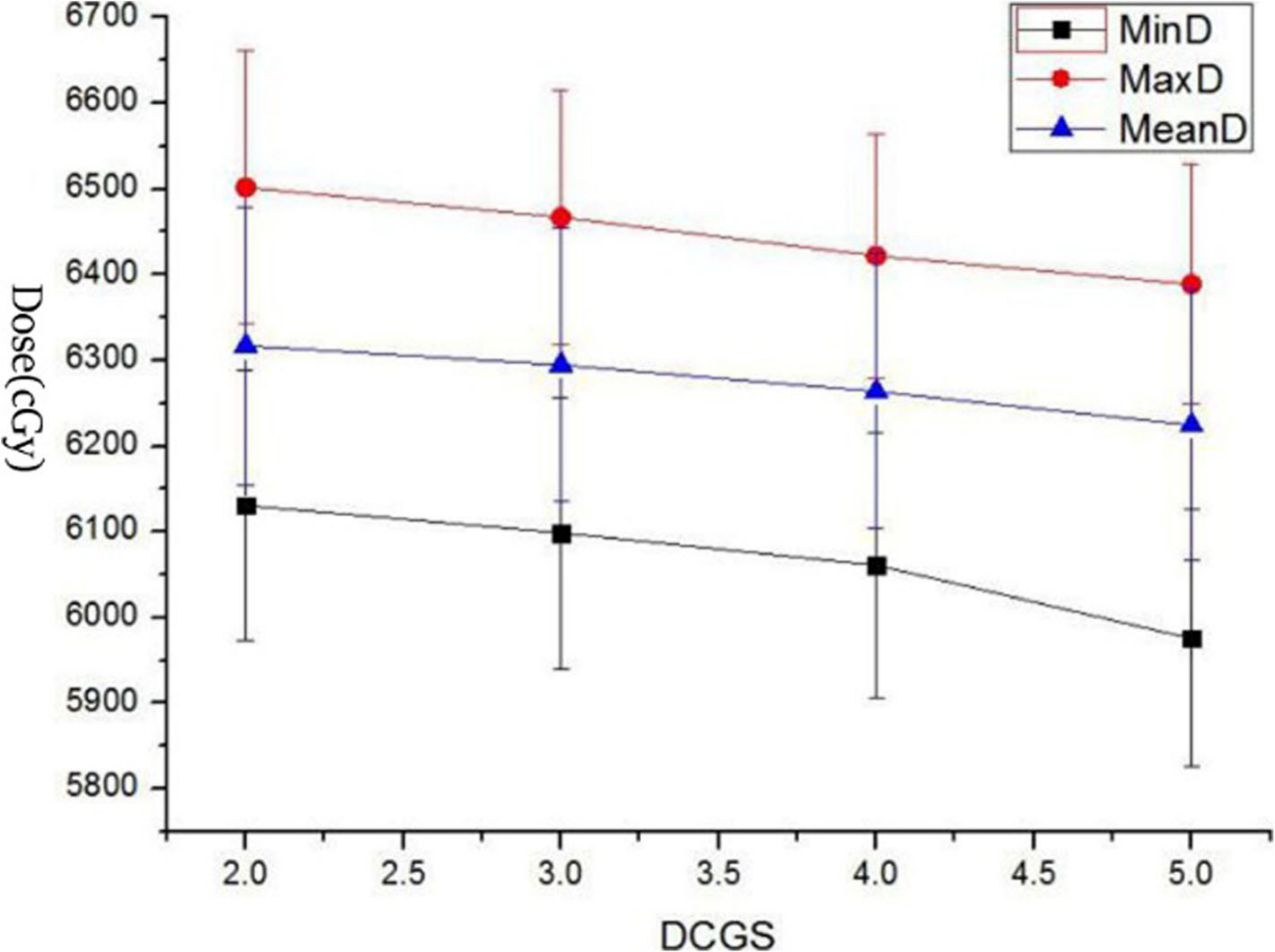
Display of MinD, MaxD and MeanD of PGTV change casused by DCGS

**Fig. 2:**
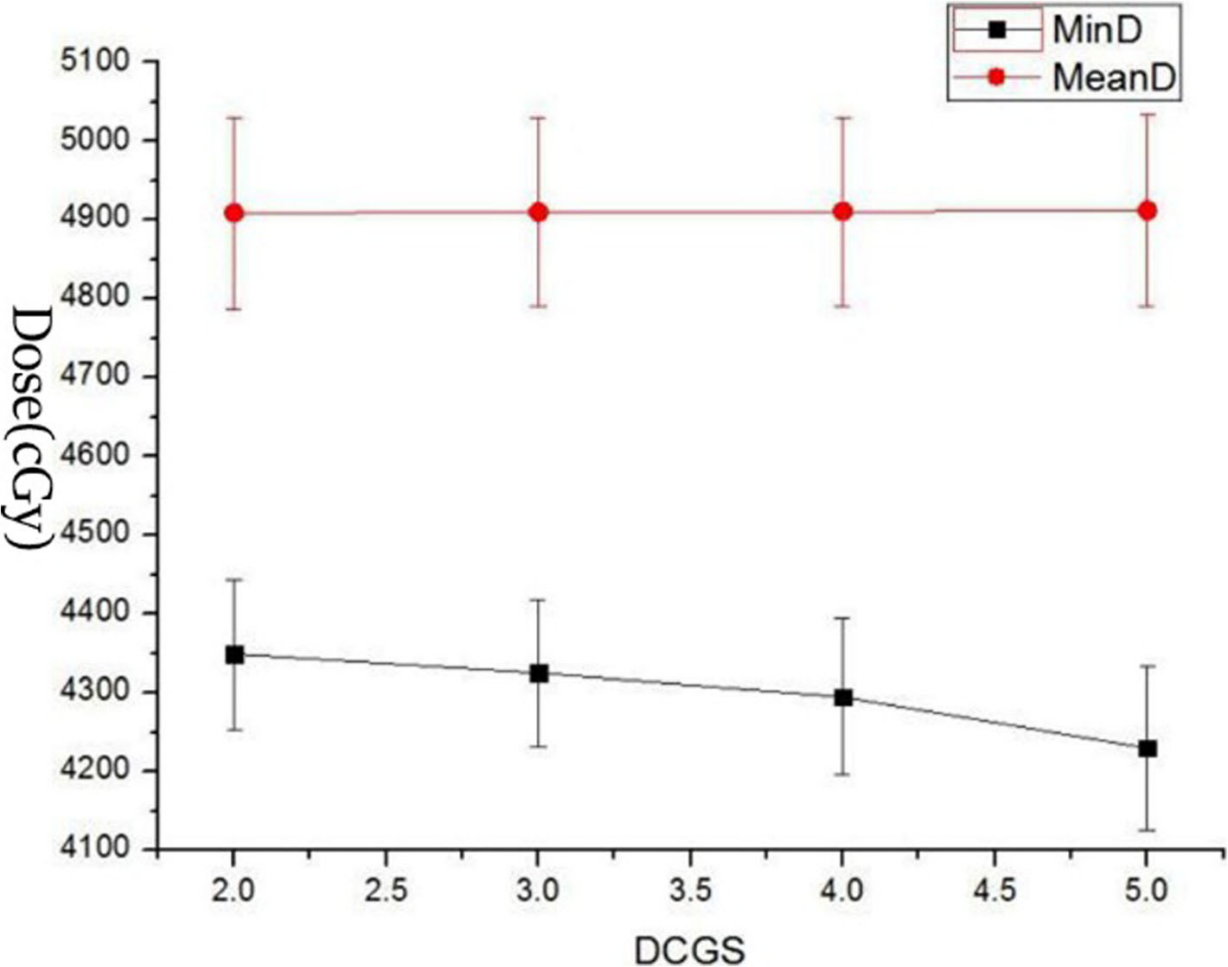
Display of MinD and MeanD of PTV change casused by DCGS

**Fig. 3:**
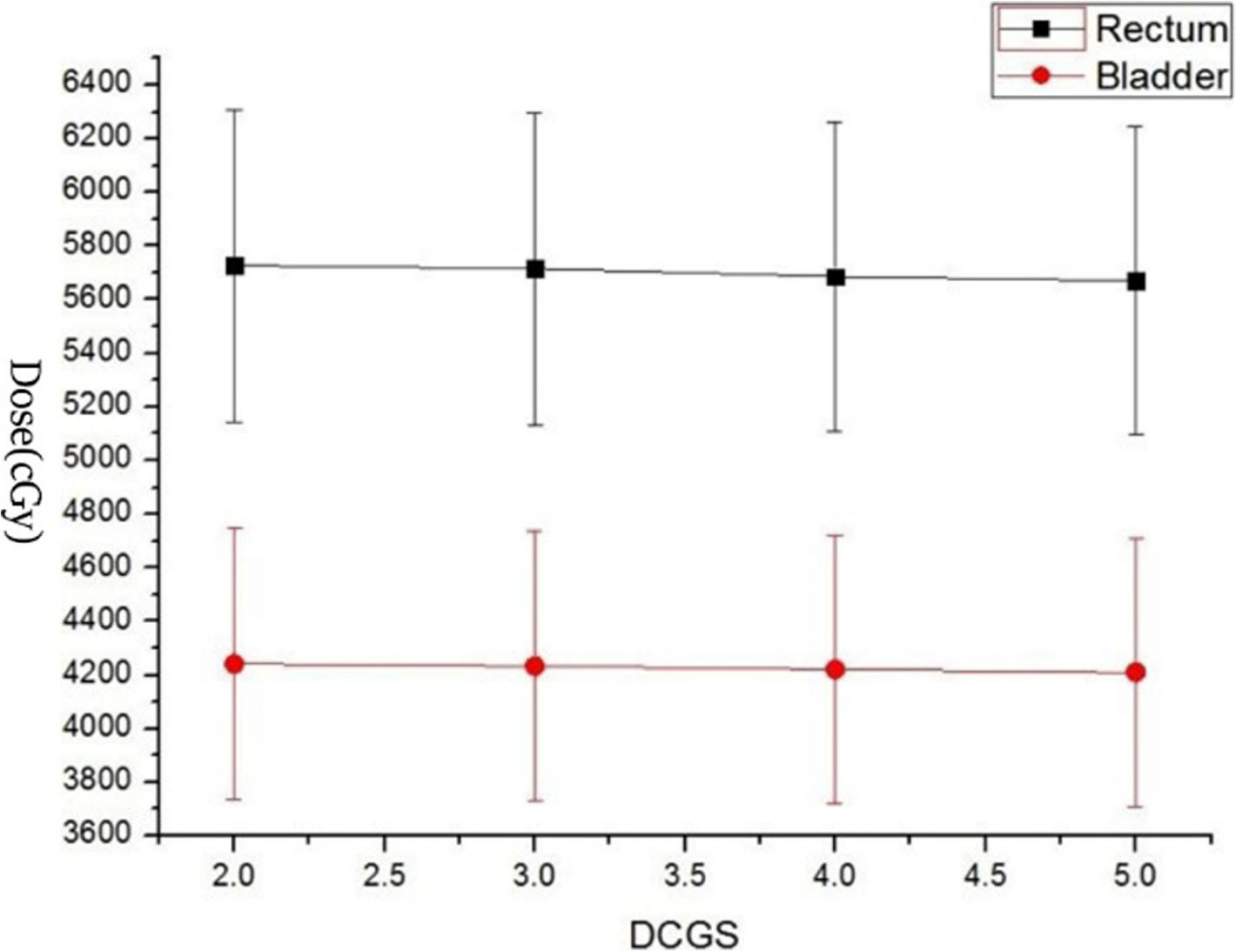
Display of MeanDs of Rectum and Bladder change casused by DCGS

**Fig. 4:**
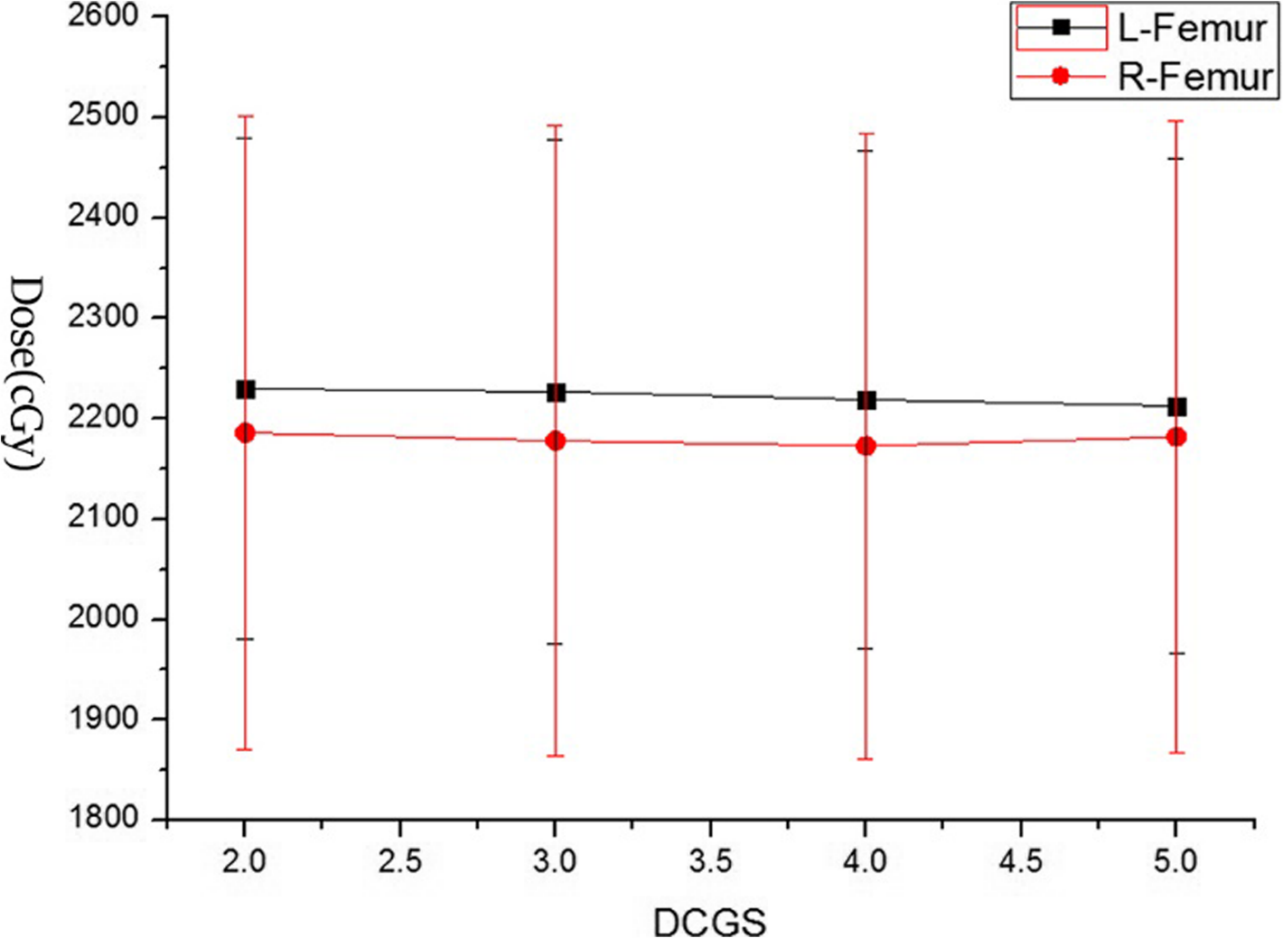
Display of MeanDs of R-Femur and L-Femur change casused by DCGS

The TPS software takes the cube formed by DCGS as the unit to collect the dose, and then generates the DVH. DCGS’s change indicated the change of “collection-dose-unit”. Key dose points on DVH of each structure with DCGS’s change were counted and analysed. As we all know, for target, minimum dose (MinD), maximum dose (MaxD) and mean dose (MeanD) were important for efficacy [34]. And for parallel organ-at-risk, MeanD was important for toxicity [35]. So, We counted on PGTV’s MinD, MaxD and MeanD, and PTV’s MinD and MeanD (because PGTV is included in PTV, the maximum dose in PTV is located in PGTV), and MeanD of Bladder, Rectum and Femurs (shown in Figs. 1, 2, 3 and 4).

**Fig. 5:**
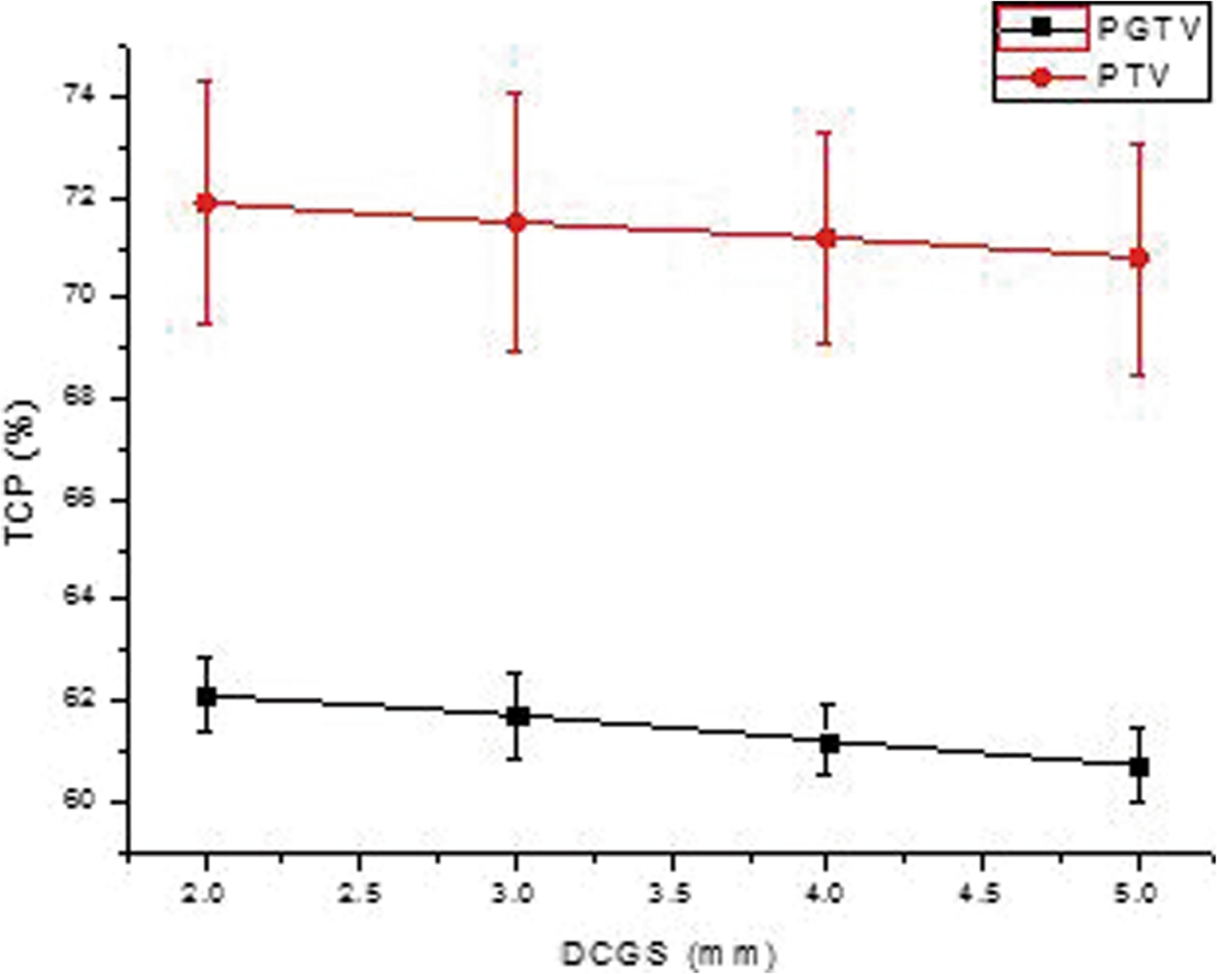
Display of TCP change casused by DCGS

**Fig. 6:**
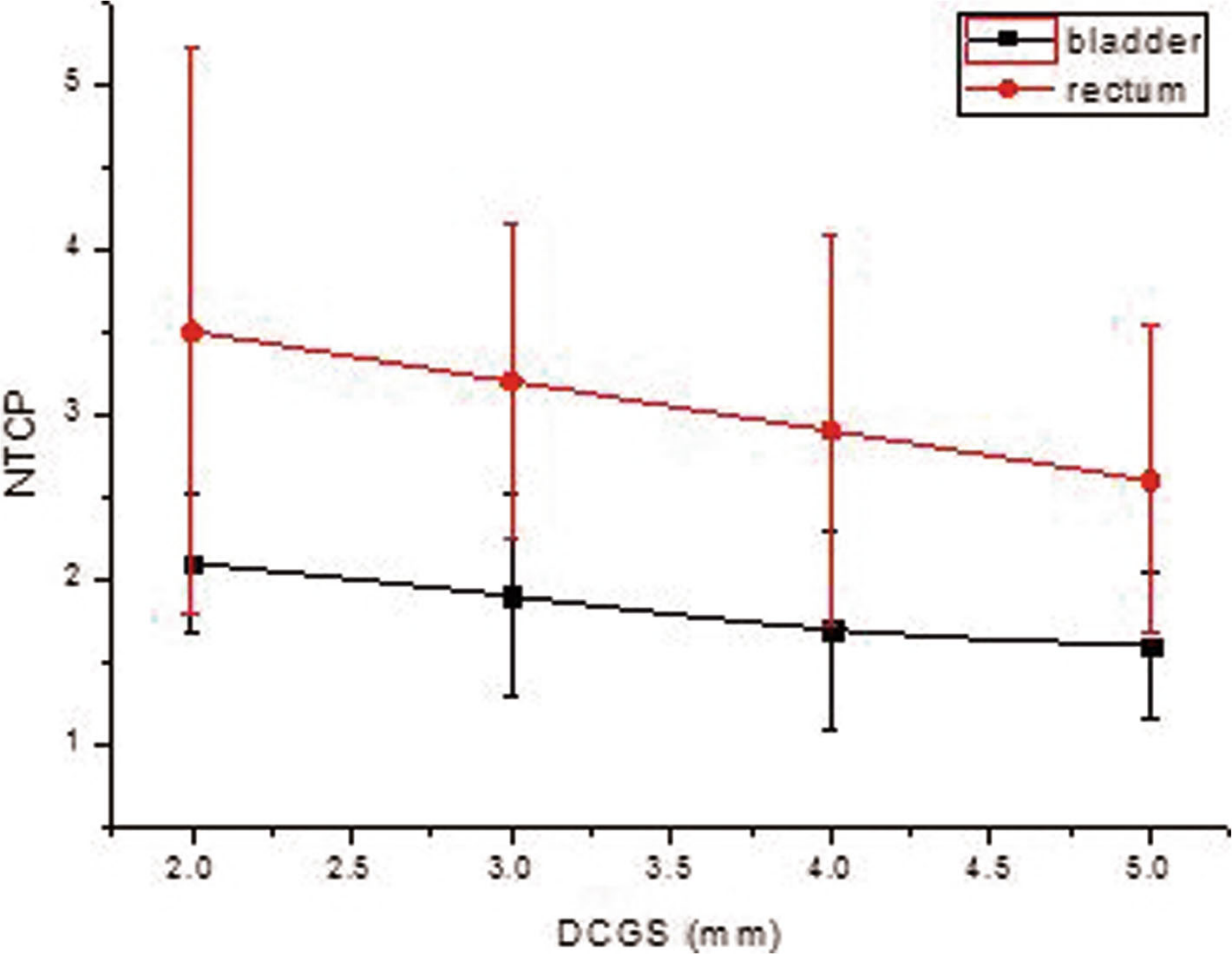
Display of NTCP change casused by DCGS

The 68 differential DVH of 17 patients’ radiotherapy plans (17*4 = 68) were exported as .txt data files. The data in the .txt file were read by MATLAB program and put into formulas (1) and (2.4) to calculate the EUD of each OAR and each target. Then TCP and NTCP were calculated using the formulas (1, 2.1). Figures drawn by Origin8.0 software were shown in Fig. 5. and Fig. 6. As shown in the figures, the influence of DCGS on the calculated value of (N) TCP was obvious, and the calculated value of (N) TCP decreased with the increase of DCGS.

### *p*-value analysis

The change of DCGS would bring about the change of each structure’s absorption dose. In order to quantify the dose change, we selected gamma analysis to analyze the difference between the dose corresponding to DCGS = 2.0 mm acting as RDS and the dose corresponding to DCGS = 3.0 mm, 4.0 mm, 5.0 mm acting as MDS, respectively.

**Fig. 7:**
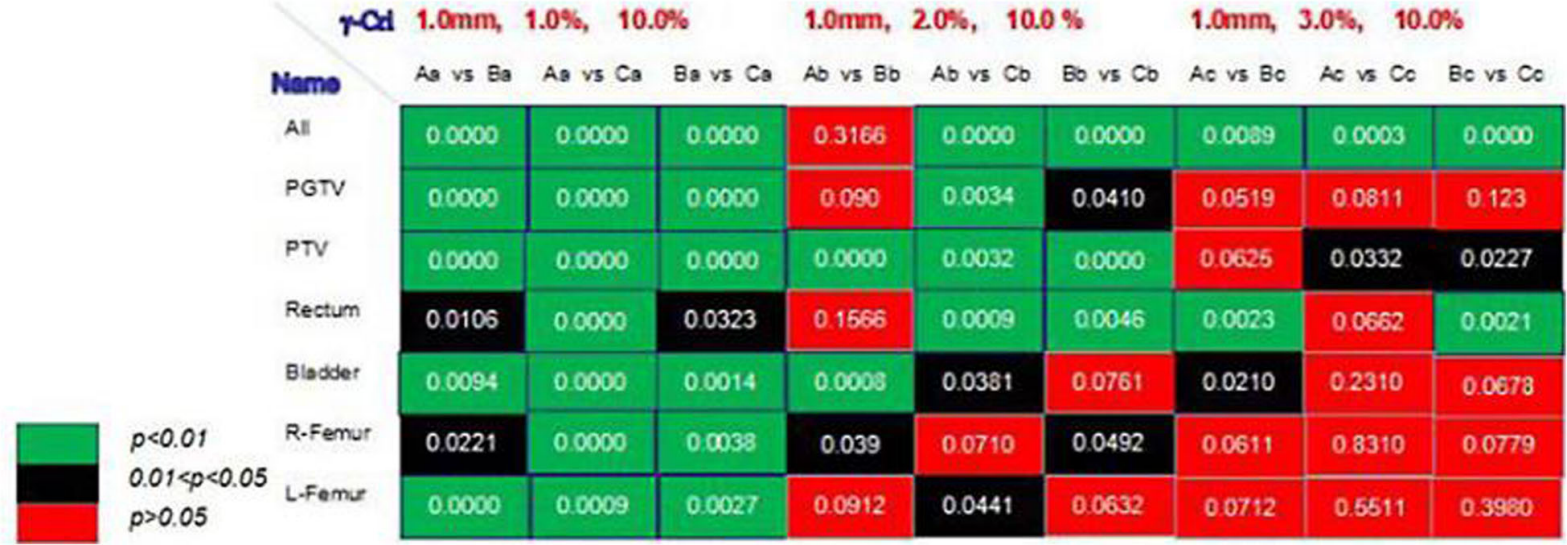
The map of every structure’s paired t test under varied gamma analysis standard. When the gamma analysis standard setting was 1.0 mm, 1.0% and the dose distributions of DCGS = 3.0, 4.0, 5.0 mm were compared with DCGS = 2.0 mm respectively and the results were grouped as Aa, Ba, Ca. When the gamma analysis standard setting was 1.0 mm, 2.0% and the dose distributions of DCGS = 3.0, 4.0, 5.0 mm were compared with DCGS = 2.0 mm respectively and the results were grouped as Ab, Bb, Cb. When the gamma analysis standard setting was 1.0 mm, 3.0% and the dose distributions of DCGS = 3.0, 4.0, 5.0 mm were compared with DCGS = 2.0 mm respectively and the results were grouped as Ac, Bc, Cc

The paired *t-*test results of each structure were shown in Fig. 7. The gamma-standard had a significant impact on the gamma-analysis’ sensitivity. When the gamma-standard was 1.0 mm, 1.0%, the difference of the results of the DCGS on dose-effect could be detected by 3Dgamma analysis (all *p* value< 0.05). With the decline of the standard, 3Dgamma analysis’ ability to detect this difference was also declining. When the standard was 1.0 mm, 3.0%, the *p* value of > 0.05 accounted for the majority. It was a high probability event that the dose difference between DCGS = 5.0 mm and DCGS = 3.0 mm (or the other two DCGSs) could not be detected by this analysis.

### Correlation of ∆(N) TCP and ∆gamma

The (N)TCPs’ and gamma values both changed with DCGS. To explore whether there was a certain correlation between these two changes, we investigated **∆**(N) TCP and**∆**gamma separately. **∆**(N) TCP was defined as the (N) TCP value when DCGS = 2.0 mm minus the (N) TCP value when DCGS was the other value. **∆**gamma was defined as 100.0% minus the gamma passing rate when the dose distribution of DCGS = 3.0 mm, or 4.0 mm, or 5.0 mm compared that of DCGS = 2.0 mm with gamma analysis. Because gamma analysis was carried out with three different standards in this paper, the **∆**gamma were divided into **∆**gamma1, **∆**gamma2 and **∆**gamma3 corresponding to 1.0 mm, 1.0%; 1.0 mm, 2.0% and 1.0 mm, 3.0%.

In order to simplify the following writing, we describe the corresponding relationship as follows.

“M” corresponding to ∆(N) TCP = (N) TCP (DCGS = 2.0 mm) -(N) TCP (DCGS = 3.0 mm) and.

∆gamma = 100.0% - gamma passing rate when DCGS = 3.0 mm compared DCGS = 2.0 mm.

“N”corresponding to ∆(N)TCP = (N) TCP (DCGS = 2.0 mm) -(N) TCP (DCGS = 4.0 mm) and.

∆gamma = 100.0% - gamma passing rate when DCGS = 4.0 mm compared DCGS = 2.0 mm.

“L”corresponding to ∆ (N)TCP = (N) TCP (DCGS = 2.0 mm) -(N) TCP (DCGS = 5.0 mm) and.

∆gamma = 100.0% - gamma passing rate when DCGS = 5.0 mm compared DCGS = 2.0 mm.

The ∆(N) TCP and ∆gamma were shown in Table 2, Table 3 and Table 4. As shown in the tables, when the calculated values of (N) TCP of the targets, the bladder and the rectum decreased with DCGS increasing, the gamma passing rate also decreased when the standard was 1.0 mm, 1.0%.

**Table 2:** Display of △TCP and △γ

*Name*	*PGTV*				*PTV*			
	*△TCP*	*△γ1*	*△γ2*	*△γ3*	*△TCP*	*△γ1*	*△γ2*	*△γ3*
M	0.40	*0.20*	*0.00*	*0.00*	*0.40*	*0.86*	*0.27*	*0.00*
*N*	*0.90*	*1.63*	*0.20*	*0.00*	*0.70*	*1.47*	*0.61*	*0.00*
*L*	*1.40*	*10.61*	*0.71*	*0.00*	*1.10*	*6.75*	*1.66*	*0.48*

**Table 3:** Display of △NTCP and △γ for the bladder and the rectum

*Name*	*Bladder*				*Rectum*			
	*△NTCP*	*△γ1*	*△γ2*	*△γ3*	*△NTCP*	*△γ1*	*△γ2*	*△γ3*
M	0.20	*0.50*	*0.12*	*0.00*	*0.30*	*0.08*	*0.16*	*0.03*
*N*	*0.40*	*1.54*	*0.69*	*0.37*	*0.60*	*2.15*	*0.40*	*0.40*
*L*	*0.50*	*4.58*	*1.22*	*0.33*	*0.90*	*2.99*	*1.42*	*0.00*

**Table 4:** Display of △NTCP and △γ for the femurs

*Name*	*L-Femur*				*R-Femur*			
	*△NTCP*	*△γ1*	*△γ2*	*△γ3*	*△NTCP*	*△γ1*	*△γ2*	*△γ3*
M	0.00	*0.02*	*0.00*	*0.00*	*0.00*	*0.17*	*0.00*	*0.00*
*N*	*0.00*	*1.07*	*0.19*	*0.01*	*0.00*	*0.75*	*0.02*	*0.00*
*L*	*0.00*	*2.93*	*0.60*	*0.08*	*0.00*	*1.70*	*0.33*	*0.00*

## Discussion and conclusion

Compared with conformal radiotherapy, intensity-modulated radiotherapy can improve the conformal degree of the target area, reduce the dose of organs at risk, and reduce the acute and late toxicity of organs [36, 37]. VMAT is a higher form of modulated radiation therapy, VMAT and IMRT have been compared in many studies [38, 39]. The publications relating to planning [40], commissioning [13], QA [41] and clinical implementation [42] have been published, which made VMAT technology spread quikly around the world. Therefore, the cervical cancer’s VMAT plans were selected as the research object in this study. The gantry and dose rate change simultaneously when a VMAT plan was executed, which makes VMAT’s QA are more complicated than IMRT [43]. It was therefore difficult to define whether the failure of a VMAT QA was possibly caused by dose calculation from TPS, dose delivery from linac, detectors of phatom, or other aspects.

It is an important basis for us to set up DCGS in the planning design to consider the efficiency of calculation under the precise of satisfying the accuracy of dose calculation. Many studies from radiation oncology deparments recommend DCGS = 2.0 mm be for the clinical requirements [44, 45]. This conclusion was the main reason why in this paper we chose DCGS = 2.0 mm as the research basis. Secondly, the low computational efficiency of DCGS = 1.0 mm makes it difficult in clinical practice, when was DCGS = 1.0 mm, the Pinnacle TPS would spend about two 2.0 h to calculate once a dose distribution for a patient.

Gamma analysis is a commonly used method to compare differences between two dose distributions, but the ability of gamma analysis to detect errors is closely related to the criteria set. Figure 7 of the “***p*-value analysis**” in this paper showed that when 1.0 mm, 1.0% was used as the standard in the gamma analysis, there was a statistical difference (all *p* value< 0.05) between any two results of gamma analysis when DGCS was 3.0 mm, 4.0 mm or 5.0 mm*vs* 2.0 mm, indicating that the gamma analysis was sensitive to changes of DCGS. However, when 1.0 mm, 3.0% was used as the standard in gamma analysis, there was mostly no significant difference (all *p* value> 0.05) between any two results of gamma analysis when DGCS was 3.0 mm, 4.0 mm or 5.0 mm*vs* 2.0 mm, and at this time the gamma analysis was not sensitive to changes of DCGS. Many studies’ results all reflected the similar conclusion of gamma-standard and gamma analysis’sensitivity [46, 47]. The difference was that these studies had set change of the standard in two dimensions (distance and dose) at the same time, for example, change the gamma_3.0%, 3.0mm_ to gamma_2.0%, 2.0mm_. When the dose standard of gamma analysis was relaxed from 1.0 to 3.0%, and those “points” that fail to pass the 1.0 mm, 1.0% standard were passed under the 1.0 mm, 3.0% standard, which meaned that the new standard lost the ability to detect dose errors in the range of 1.0–3.0%. At the same time, we could also get from the results that the majority of the dose “calculated value” changes as DCGS from 2.0 mm to 5.0 mm were < 3.0%.

However, the situation was different when the (N) TCP biological mathematical model was used to detect these dose changes, and theoretically any dose change caused by DCGS could be represented in the value of (N) TCP as long as the value of (N) TCP was accurate enough. So, when we changed DCGS we got the trend of (N) TCP and ∆ (N) TCP, even though ∆(N) TCP was not a big value. In the study, ∆NTCP of the Femurs was always 0.00 in Table 4. It was not because NTCP did not change, but because the value was omitted because of too small, which was cause by parameters’ value of NTCP model.

**Fig. 8:**
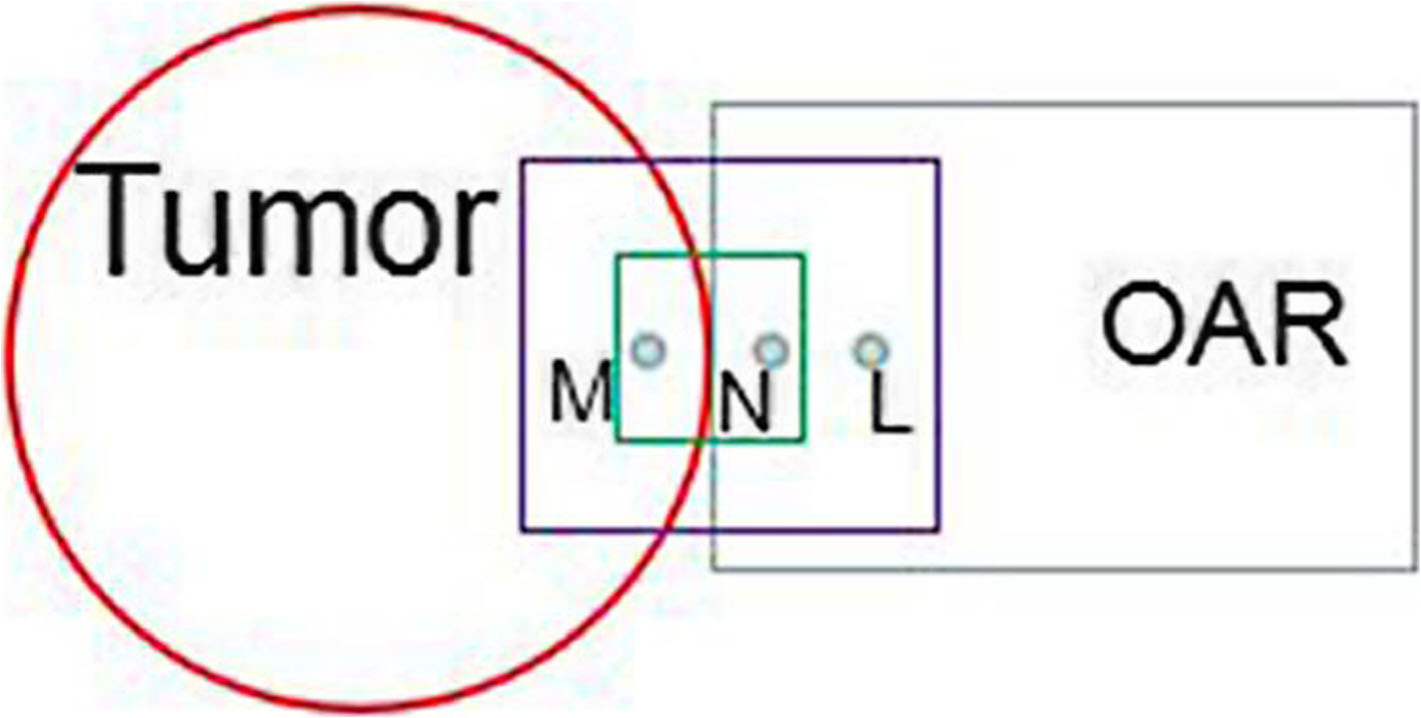
Schematic diagram of dose collection by different DCGS

The TCP and NTCP were expected to be obtained by studying the targets’ and OARs’ physical dose, because the two formers were of greater clinical significance. So this paper studied the relationship between ∆(N) TCP and ∆gamma. With the DCGS becoming larger, the relatively-lower-dose in the normal tissues located around the target was more calculated into the target, so the overall dose of the target decreased and the TCP decreased. As shown in Fig. 8, in most cases, D_M_ (absorbed dose at M point) > D_N_ (absorbed dose at N point) > D_L_ (absorbed dose at L point), When DCGS was small (corresponding to the green box), it collected D_M_ and D_N_. As.

the DCGS became larger (corresponding to the purple box), it collected a lower dose of D_L_ than the D_N_ dose. The focused irradiation mode of radiotherapy determines the general trend of dose decrease from the target area to the periphery, with the highest dose in the target. In this paper, we investigated the OARs, bladder, rectum, and femurs were the organs of adjacent to the targets, as the DCGS became larger, their relatively-high-dose was “deprived” by the targets, and the dose “deprived” by them from their surrounding was relatively-high-dose, so the DCGS became larger, their overall doses were falling, and their NTCPs were falling.

Some previous studies reported the relationship between DCGS and gamma passing rate, and the results showed that DCGS = 1.0–4.0 mm could meet most clinical needs [48, 49]. However, from the perspective of plan evaluation in this paper, large DCGS made us make a “low evaluation” for the patient’s radiation dose, and the patient’s actual radiation dose was larger than the dose we “saw”.

The 3Dgamma analysis and bio-mathematical model can be used to analyze the effect of DCGS on the planned dose, and the former’s detection ability has a lot to do with the designed standard, and the latter’s capability is related to the parameters and calculated accuracy of the latter.

## Data Availability

The datasets generated and/or analysed during the current study are not publicly available due to the General Data Protection Regulation (GDPR) but are available from the corresponding author on reasonable request.

## Abbreviations

DCGS: Dose calculation grid size
PD: Planned dose
DVH: Dose-volume histogram
RDS: Reference data set
MDS: Measurement data set
TCP: Tumor control probability
NTCP: Normal tissue complication probability
TPS: Treatment planning system
OAR: Organ-at-risk
DD: Dose distribution
IMRT: Intensity modulated radiation therapy
VMAT: Volumetric modulated arc therapy
PTV: Planning target volume
PGTV: Planning gross tumor volume
QA: Quality assurance
